# Comment on “Meta‐Analysis of Cost‐Effectiveness” by Bang and Zhao: Combinability, Joint Modeling, and a Two‐Stage Framework for Cost‐Effectiveness Analysis Synthesis

**DOI:** 10.1002/sim.70575

**Published:** 2026-05-19

**Authors:** Gian Luca Di Tanna, Niklaus Meier, Matthias Schwenkglenks

**Affiliations:** ^1^ University of Applied Sciences and Arts of Southern Switzerland – SUPSI Lugano Switzerland; ^2^ Institute of Health Economics and Health Policy Bern University of Applied Sciences Bern Switzerland; ^3^ Health Economics Facility, Department of Public Health University of Basel Basel Switzerland

**Keywords:** combinability, cost‐effectiveness analysis, data harmonization, incremental cost‐effectiveness ratio, meta‐analysis, systematic review of economic evaluation

## Abstract

Bang and Zhao address an important methodological gap by proposing meta‐analytic methods for cost‐effectiveness analysis (CEA) based on separate pooling of incremental costs (ΔC) and incremental effectiveness (ΔE), with summaries displayed on the cost‐effectiveness plane. We share the aim of expanding the toolkit for CEA synthesis; however, we believe that several issues need clarification before the proposal can serve as a general blueprint. First, the two motivating examples appear not to satisfy the key combinability criteria described by Shields and Elvidge. Second, the proposal does not incorporate the data harmonization steps recommended for the meta‐analysis of economic evaluations by Bagepally et al. Third, separate univariate pooling does not retain the within‐study association between ΔC and ΔE, which complicates the joint interpretation of the pooled ICER and its uncertainty. As a possible way forward, we propose a two‐stage framework in which a structured combinability assessment and data harmonization precede any quantitative synthesis, and discuss why joint modeling of (ΔC, ΔE) is preferable to separate univariate pooling. In our view, such a framework provides a more defensible route to pooled ICERs, joint uncertainty summaries, and decision‐relevant quantities such as cost‐effectiveness acceptability curves (CEACs).

## 1 Introduction

The scarcity of formal meta‐analytic methods for cost‐effectiveness analysis (CEA) is a methodological gap, and Bang and Zhao's contribution to this field is welcome [1]. Their ratio‐weighting (Scenario 1) and inverse‐variance‐weighting (Scenario 2) estimators for ΔC and ΔE, together with the bootstrap wedge confidence arc for the incremental cost‐effectiveness ratio (ICER), move the discussion in a useful and accessible direction. We also agree with the broader motivation of the paper, namely that methods for synthesizing cost‐effectiveness evidence remain underdeveloped relative to the centrality of such evidence in decision‐making.

Notwithstanding this, we have several concerns. A framework meant to guide future applications needs examples that are plausibly commensurable, explicit attention to the harmonization steps required before pooling, and an inferential structure that respects the joint nature of cost and effect outcomes. In our view, these elements need to be more fully developed before the proposed approach can serve as a general blueprint. In this comment, we detail our concerns and sketch a possible way forward, namely a more explicit two‐stage architecture in which the feasibility of pooling is assessed first, and joint modeling is performed only thereafter.

## 2 Overall Critique

Our main concerns can be summarized as follows:

1. The two motivating examples do not appear to satisfy the key combinability criteria for CEA synthesis [2];
2. The proposal does not incorporate the data harmonization steps that are generally needed before costs can be meaningfully synthesized across settings [3];
3. Separate univariate pooling of ΔC and ΔE does not preserve their within‐study association, making joint interpretation of the pooled ICER and its uncertainty more difficult;
4. For Reasons 1–3, the proposed approach, as illustrated, lacks the external validity required to support real‐world decision‐making.

## 3 The Motivating Examples Raise Combinability Concerns

Shields and Elvidge describe 10 challenges that should be considered before CEA meta‐analysis is attempted, including heterogeneity in outcomes, cost perspective, currency and price year, time horizon, discounting, and structural differences between trial‐based and model‐based evaluations [2]. Bang and Zhao cite this work approvingly, while also noting that their own analyses are intended as a statistical demonstration rather than a full assessment of comparability [1]. We acknowledge that this distinction is important. However, as these examples are used to motivate a general framework, the extent to which they satisfy the prerequisites for pooling becomes central.

In the first example, based on Tricco et al. [4], effectiveness is reported on at least four different scales: QALYs, ulcer‐free weeks, ulcer‐free months, and patient‐years. This alone raises a substantial obstacle to direct synthesis. The studies also span different wound types, mix trial‐based and model‐based evaluations, and operate over different time horizons. In the second example, based on Dewa et al. [5], costs from the Netherlands, Sweden, and Canada are pooled without standardization to a common currency and price year. The same set of studies also mixes societal and payer perspectives and differs in analytic design and time horizon. In light of the criteria described by Shields and Elvidge [2], both examples should rather be viewed as illustrations of why a feasibility screen is needed than as empirical demonstrations of pooling appropriateness.

The consequence is not simply a limitation in generalizability. Even a statistically well‐behaved estimator can yield a pooled quantity with uncertain clinical or economic meaning if the underlying inputs are not commensurable. The choice of estimator is moot if the studies to be pooled are not comparable; combinability must therefore be established before any synthesis method is applied.

## 4 Data Harmonization Should Be Part of the Framework

Bagepally et al. provide a practical framework for harmonizing economic evaluation data prior to meta‐analysis [3]. Their recommendations include conversion of costs to a common purchasing‐power‐parity adjusted currency, alignment of price years using country‐specific inflation data, and explicit handling of missing variance information. These steps are required to ensure that the quantities entering a synthesis are measured on a common and interpretable scale.

The Dewa example illustrates this clearly [5]. We recognize that Bang and Zhao were working with a published systematic review and that the scope for retrospective harmonization was necessarily limited in that setting. Nevertheless, the example highlights a more general point: any prospective application of the proposed framework should treat harmonization as a prerequisite rather than an optional step. In the Dewa example, costs from the Netherlands, Sweden, and Canada are pooled without standardization to a common currency and price year, across studies that differ in perspective and time horizon. Scenario 1 in Bang and Zhao's framework is useful in allowing synthesis when standard errors are unavailable; a companion harmonization procedure, of the kind described by Bagepally et al. [3], is needed to ensure that the cost quantities themselves are comparable before pooling is attempted.

## 5 Separate Univariate Pooling Does Not Retain the Joint Cost‐Effectiveness Structure

The ICER is an important and widely used summary in CEA, and we have no objection to its use as a target of synthesis when the contributing studies are sufficiently comparable. Our concern lies instead with the route by which Bang and Zhao obtain it. Incremental costs and incremental effects arise from the same comparison of two or more interventions and are typically correlated within any given study: a treatment that is more effective will often, but not always, also be more costly, and the strength of this association varies across studies. If ΔC and ΔE are first pooled separately by univariate meta‐analysis, this association is no longer carried through to the final joint summary. Ignoring it mischaracterizes the uncertainty around the decision problem as a whole, including the ICER.

When two uncorrelated confidence intervals for ΔC and ΔE are combined, their joint uncertainties form a rectangle on the incremental cost‐effectiveness plane. This matters for interpretation: a rectangle formed from the marginal intervals for pooled ΔC and pooled ΔE is not, in general, a joint uncertainty region unless restrictive conditions hold. Likewise, while the bootstrap wedge is an informative graphical device for the ICER slope, it does not by itself recover the dependence structure lost when the pooled cost and effect components are estimated separately. The problem becomes more acute when pooled ΔE is close to zero, as in the Dewa example [5], because the ICER then becomes highly unstable and its sign is difficult to interpret. Negative ICERs, as is well known, may reflect either dominance or inferiority depending on the quadrant of the cost‐effectiveness plane.

A decision‐focused synthesis is better served by an approach that models (ΔC, ΔE) jointly and propagates this joint uncertainty through to the ICER and related summaries.

## 6 A Constructive Two‐Stage Framework

One way to consolidate these concerns is to make the analytical logic an explicit two‐stage process.

In Stage 1, studies would first be screened for feasibility of pooling using a structured checklist informed by Shields and Elvidge [2], covering population comparability, time horizon, discounting, effectiveness metric, and analytic design. For studies that pass this screen, cost data would then be harmonized following Bagepally et al. [3], including conversion to a common purchasing‐power‐parity adjusted currency and alignment of price years. Only studies that are both sufficiently commensurable and harmonized would proceed to quantitative synthesis.

In Stage 2, the retained studies would be analyzed jointly, treating (ΔC, ΔE) as a correlated pair rather than pooling incremental costs and incremental effects separately. A Bayesian bivariate random‐effects model [6] offers a principled route to preserving the within‐study association, propagating joint uncertainty through to the pooled ICER, and supporting the construction of credible regions on the cost‐effectiveness plane and cost‐effectiveness acceptability curves across a range of willingness‐to‐pay values. A full specification of such a framework, including prior choice, transformation strategies for skewed cost distributions, and the construction of decision‐relevant summaries, is the subject of ongoing work by the authors.

Applying this logic to the Tricco review [5] illustrates the consequence directly. Restricting synthesis to the more comparable trial‐based QALY studies changes both the evidential basis and the resulting decision summaries materially, relative to pooling the full heterogeneous set. Combinability is not a procedural checkbox; it determines what question the synthesis actually answers.

## 7 Conclusions

Bang and Zhao deserve credit for drawing attention to an area where methodological development is clearly needed. Our view, however, is that progress in CEA meta‐analysis will be most convincing when it proceeds in two linked steps: first, a transparent assessment of whether studies are combinable—including data harmonization where required—and second, a joint modeling strategy that respects the bivariate structure of incremental costs and effects. The value of such a framework lies not only in its statistical properties, but in its capacity to produce decision‐relevant outputs—pooled ICERs, joint uncertainty regions, and CEACs—that can inform decision‐making. This requires that the contributing studies possess sufficient external validity which, in turn, makes combinability an indispensable criterion, not a preliminary formality.

Framed in this way, the paper may be seen not as a finished template for practice, but as a useful prompt for a more systematic framework, one in which combinability is assessed first, and joint modeling is applied only where the resulting pooled quantities can be meaningfully interpreted and used to inform decision‐making.

## Funding

The authors have nothing to report.

## Conflicts of Interest

The authors declare no conflicts of interest.

## Data Availability

Data sharing not applicable to this article as no datasets were generated or analysed during the current study.
